# HIV-1 designed to use different tRNA^Gln ^isoacceptors prefers to select tRNA^Thr ^for replication

**DOI:** 10.1186/1743-422X-3-80

**Published:** 2006-09-26

**Authors:** Meng Li, Peter G Eipers, Na Ni, Casey D Morrow

**Affiliations:** 1 Department of Cell Biology, University of Alabama at Birmingham, 35294-0024 Birmingham, AL, USA

## Abstract

**Background:**

Previous studies have shown that infection with human immunodeficiency virus type 1 (HIV-1) causes acceleration of the synthesis of glutamine tRNA (tRNA^Gln^) in infected cells. To investigate whether this might influence HIV-1 to utilize tRNA^Gln ^as a primer for initiation of reverse transcription, we have constructed HIV-1 proviral genomes in which the PBS and the A-loop region upstream of the PBS have been made complementary to either the anticodon region of tRNA^Gln,1 ^or tRNA^Gln,3 ^and 3' terminal 18 nucleotides of each isoacceptor of tRNA^Gln^.

**Results:**

Viruses in which the PBS was altered to be complementary to tRNA^Gln,1 ^or tRNA^Gln,3 ^with or without the A-loop all exhibited a lower infectivity than the wild type virus. Viruses with only the PBS complementary to tRNA^Gln,1 ^or tRNA^Gln,3 ^reverted to wild type following culture in SupT1 cells. Surprisingly, viruses in which the PBS and A-loop were complementary to tRNA^Gln,1 ^did not grow in SupT1 cells, while viruses in which the PBS and A-loop were made complementary to tRNA^Gln,3 ^grew slowly in SupT1 cells. Analysis of the PBS of this virus revealed that it had reverted to select tRNA^Thr ^as the primer, which shares complementarity in 15 of 18 nucleotides with the PBS complementary to tRNA^Gln,3^.

**Conclusion:**

The results of these studies support the concept that the HIV-1 has preferred tRNAs that can be selected as primers for replication.

## Background

HIV-1 reverse transcription is initiated with the extension of the cellular tRNA that is bound to a specific sequence on the viral RNA genome known as the primer-binding site (PBS) [1-3]. The PBS is an 18-nucleotide sequence located near the 5' end of viral RNA that is complementary to the 3' terminal nucleotides of the primer tRNA used for initiation [3]. HIV-1 specifically selects tRNA^Lys,3 ^from the intracellular milieu to be used as the primer for initiation of reverse transcription [4,5]. The mechanism of how HIV-1 specifically selects tRNA^Lys,3 ^from the intracellular milieu is not completely understood. Previous studies have established that tRNA^Lys,3 ^as well as tRNA^Lys1,2 ^are enriched in HIV-1 virions [6-8]. The Gag-Pol polyprotein of HIV-1 is responsible, in part, for this enrichment of tRNA^Lys1,2,3 ^into the virions [4,6,8]. Studies have also demonstrated that lysl tRNA synthetase can specifically interact with HIV-1 Gag to facilitate incorporation of tRNA^Lys1,2,3 ^into HIV-1 virions [9-11]. Once this complex is incorporated into virions though, it is not clear how and why tRNA^Lys,3 ^is specifically utilized as the primer for initiation of reverse transcription.

Previous studies from our lab and others have taken a genetic approach to understanding elements of HIV-1 primer selection [12-14]. For these studies, we have mutated the PBS to be complementary to tRNAs other than tRNA^Lys,3^. In general, mutation of the PBS to be complementary to other tRNAs, including tRNA^Lys1,2^, results in a virus that can transiently utilize the specific tRNA but most of the time reverts back to rapidly utilize tRNA^Lys,3 ^following *in vitro *culture [12-14]. Stabilization of alternative tRNAs use has been accomplished through additional mutations upstream in the U5 region designated as the A-loop, which is complementary to the anticodon region of tRNA^Lys,3 ^[15-19]. For some, but not all tRNAs, mutation of the A-loop region as well as the PBS to be complementary to the anticodon and 3' terminal nucleotides, respectively, of the tRNA allows this tRNA to be stably utilized by HIV-1 as a primer for reverse transcription. Using this strategy, we have generated viruses which stably utilized tRNA^Lys1,2^, tRNA^Met^, tRNA^His^, and tRNA^Glu ^[15-19]. A recent study has also found that HIV-1 can be forced to use tRNA^Lys1,2 ^if mutations are made complementary to nucleotides in the TϕC loop of tRNA^Lys1,2 ^in a second region upstream of the PBS, called the primer activation site [20].

All viruses that utilize alternative tRNAs do not replicate as efficiently as the wild type virus that utilizes tRNA^Lys,3^. This result has lead to the speculation that the availability of tRNA for primer selection might not be the same for all tRNAs. To test this it will be necessary to alter the levels of individual tRNA isoacceptors in cells. However, it is difficult to modulate the levels of tRNA in mammalian cells without leading to toxicity. Previous studies by Kuchino et al. though have found that the levels of a natural glutamine suppressor tRNA which exists as a minor species of glutamine tRNA (tRNA^Gln,3^) in normal cells is increased in murine leukemia virus (MuLV) infected cells [21,22]. In follow up studies, Muller et al. found that although the amount of the suppressor tRNA^Gln,3 ^was only 6% of the major glutamine tRNA^Gln,1 ^levels the amount of suppressor increased almost 20 fold while the levels of non-suppressor tRNA^Gln,1 ^remained the same in cells infected with MuLV or HIV-1 [23,24]. Since the levels of a particular tRNA (tRNA^Gln,3^) increase following infection with HIV-1, it might be possible to force HIV-1 to use this isoacceptor of tRNA^Gln ^as a primer for replication. To test this, we created viruses in which the PBS is complementary to the minor and major species of tRNA^Gln^. We also constructed viruses which contain additional mutations in the A-loop regions to determine if this will affect the stable use of these tRNAs as primers for HIV-1 reverse transcription. Results of our study show that these viruses with the PBS complementary to either tRNA^Gln ^species were unstable and rapidly reverted back to utilize tRNA^Lys,3^. Inclusion of the A-loop complementary to the anticodon of tRNA^Gln,3 ^resulted in a virus that did not revert to utilize tRNA^Lys,3 ^but selected an unexpected tRNA, tRNA^Thr^. The results of these studies suggest that certain tRNAs are favored by HIV-1 for the selection as a primer for initiation of reverse transcription.

## Results

### Construction of HIV-1 proviral genomes with PBS and A-loop complementary to tRNA^Gln^

**Figure 1:**
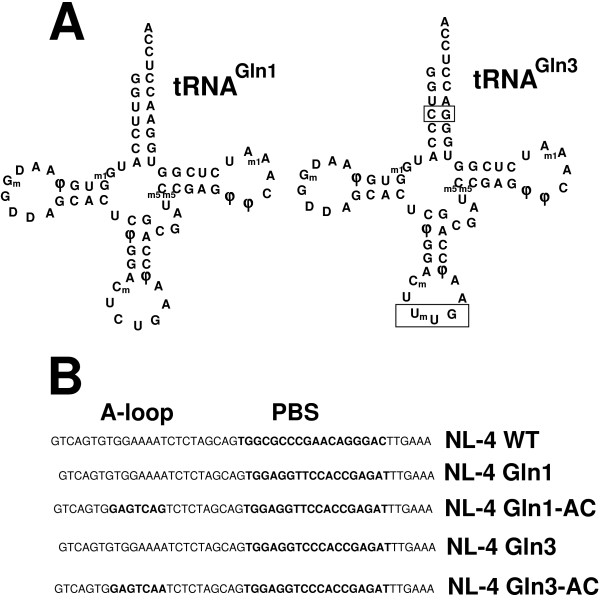
**tRNA^Gln ^and mutated proviral genomes**. **Panel A**. Cloverleaf structure of tRNA^Gln,1 ^and tRNA^Gln,3^. tRNA^Gln,1 ^(major) and tRNA^Gln,3 ^(minor) are depicted. The tRNAs differ in the nucleotides within the PBS (boxed) as well as nucleotides in the anticodon region (boxed). The modified nucleotides are noted. The structures are taken from Kuchino et al. [22]. **Panel B**. Modifications in the NL-4 proviral genome. NL-4 WT refers to the wild type NL-4 genome with the PBS and A-loop complementary to tRNA^Lys,3^. NL-4-Gln1 refers to a modified proviral genome in which the PBS was modified to be complementary to the 3' terminal nucleotides of tRNA^Gln,1^. NL-4 Gln1-AC also contains a PBS complementary to the 3' terminal nucleotides of tRNA^Gln,1 ^with additional modifications of the A-loop region (GAGTCAG) noted in bold. NL-4-Gln3 is an HIV-1 with the PBS modified to be complementary to the 3' terminal 18-nucleotides of tRNA^Gln,3^. Note that the PBS is nearly identical with the exception of the T to C change in the PBS. NL-4 Gln3-AC refers to an HIV-1 in which the PBS was modified to be complementary to tRNA^Gln,3 ^with additional modification in the A-loop region consisting of GAGTCAA which is complementary to the anticodon region of tRNA^Gln3^.

To determine if HIV-1 can utilize tRNA^Gln ^as a primer for reverse transcription, we mutated the PBS to be complementary to a 3' terminal nucleotide of tRNA^Gln^. The major isoacceptor for tRNA^Gln ^(tRNA^Gln,1^) has an anticodon CUG. A second tRNA^Gln ^has an anticodon UUG and is referred to as the minor tRNA^Gln ^or tRNA^Gln,3 ^[21,22] (Figure 1A). Previous studies have shown that in HIV-1 infected cells, the levels of tRNA^Gln,3 ^are increased 20 fold over that of uninfected cells [23]. The 3' terminal nucleotides of tRNA^Gln,1 ^and tRNA^Gln,3 ^differ only by a single nucleotide (Figure 1B). We have also constructed two additional proviruses in which the A-loop region of HIV-1 was mutated to correspond to the anticodon sequences of tRNA^Gln,1 ^and tRNA^Gln,3^, respectively (Figure 1B).

### Characterization of mutant HIV-1

The first step in the characterization of HIV-1 with the PBSs alone or PBSs in combination with A-loop modifications to be complementary to tRNA^Gln ^was to determine the effects on the infectivity of viruses following transfection. For these studies, we transfected the proviral genomes into 293T cells and assayed the supernatants for infectious virus using the JC53βL assay. We also determined the amounts of virus in the supernatants by using a p24 antigen capture ELISA. The infectivity of the viruses is represented as the amount of infectious units divided by the p24 levels. Previous studies from our laboratory have shown that for the most part, mutations within the PBS of HIV genome results in viruses that exhibit infectivity approximately 20% (or lower) of the wild type virus [25]. Similar results were found for viruses in which the PBS was made complementary to tRNA^Gln,1 ^or tRNA^Lys,3^. No significant differences were observed between viruses with the PBS alone complementary to tRNA^Gln ^and viruses with the PBS and A-loop complementary to tRNA^Gln^. The virus with a PBS and A-loop complementary to tRNA^Gln,1 ^though had the lowest infectivity, approximately 10% of the wild type virus and half as much as the other viruses in which the PBS was altered to be complementary to tRNA^Gln,3 ^(data not shown).

**Figure 2:**
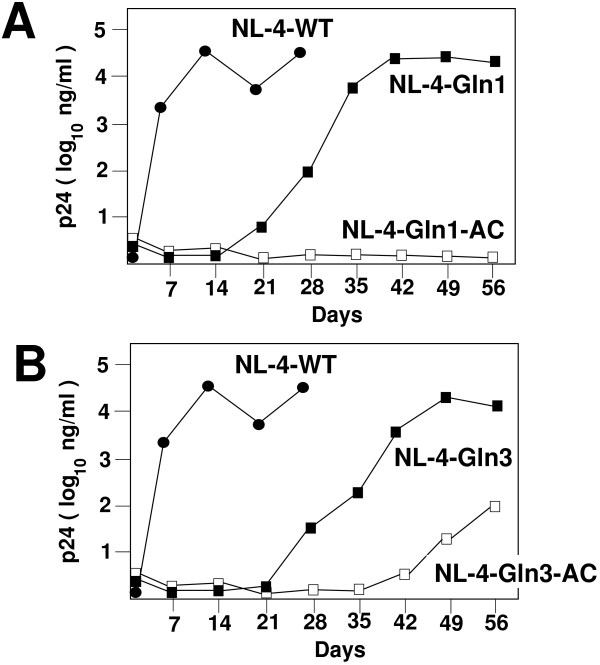
**Replication of HIV with PBS and A-loop complementary to tRNA^Gln^**. **Panel A**. Replication of wild type and viruses with PBS complementary to tRNA^Gln,1^. Infections were established in SupT1 cells with equal amounts of virus as determined by infectious units. p24 antigen was then assayed in the culture supernatants at weekly intervals following initiation of the experiment. Values for the wild type virus increased to greater than 10^4 ^nanograms/ml by approximately 14 days following initiation of the infection. The cultures were terminated at day 28. Viruses derived from NL-4-Gln1 and NL-4-Gln1-AC were carried out to approximately 56 days post initiation of culture. Note that viruses derived from NL-4-Gln1-AC did not grow, as evidenced by p24 antigen that were near the levels of mock infected cells. Cultures were terminated at day 56. Data is representative from three independent experiments. **Panel B**. Replication of viruses with the PBS complementary to tRNA^Gln,3^. The replication of the wild type virus is depicted. Cultures initiated with viruses derived from NL-4-Gln3 and NL-4-Gln3-AC were monitored over 56 days of culture. The viruses derived from NL-4-Gln3 eventually reached levels approximating that of the wild type virus by day 42 through 56. Viruses derived from NL-4-Gln3-AC demonstrated a slow and gradual increase reaching levels approximately 1/100 of that of the wild type virus at the time of termination of the culture (day 56). Data is representative of three independent experiments.

We next analyzed the replication of these viruses in SupT1 cells. Infections were established with equal amounts of infectious virus and replication was monitored by analysis of p24 in the culture supernatant. The wild type virus demonstrated a rapid increase in p24 antigen in the culture supernatant, peaking at approximately 14 days following initiation of the infection; the cultures for the wild type virus were halted at day 28 post initiation of culture. In contrast, viruses in which just the PBS alone was made complementary to tRNA^Gln,1 ^or tRNA^Gln,3 ^exhibited slower infection compared to the wild type. The p24 levels in the culture supernatants increased slowly, reaching a maximum at days 35 to 49 post initiation of culture. The final levels of p24 antigen detected in the culture supernatants from these viruses were similar to those of the wild type virus (Figure 2A). Viruses in which the PBS and A-loop were made complementary to tRNA^Gln,1 ^or tRNA^Gln,3 ^had considerably different replication profiles compared to the viruses with mutations in the PBS alone. Viruses with the PBS and A-loop complementary to tRNA^Gln,1 ^showed no increase in p24 antigen culture over the period examined (56 days of *in vitro *culture), indicating that the virus with this mutation in the PBS and A-loop did not undergo detectable replication and re-infection. In contrast, viruses with the PBS and A-loop complementary to tRNA^Gln,3 ^did replicate and eventually demonstrated an increase in p24 antigen during the 56 day culture period (approximately 100 fold over the starting amount of virus (p24 antigen) (Figure 2B).

We utilized PCR to amplify the U5-PBS region from integrated proviruses found in cellular genomic DNA to identify the PBS of viruses following *in vitro *culture. We analyzed cellular DNA obtained at day 42 from cultures infected with viruses in which the PBS alone was mutated to be complementary to tRNA^Gln,1 ^or tRNA^Gln,3 ^(Table I). In both instances, we found that analysis of U5-PBS obtained from viruses at 42 days post initiation of culture, which corresponded to the time at which there was a rise in p24 antigen, resulted in some of the viruses containing PBS complementary to the starting tRNA^Gln^. Surprisingly, the major PBS recovered from analysis of both viruses was complementary to tRNA^Thr^, indicating both viruses had switched their preference from tRNA^Gln ^to tRNA^Thr^. By day 56, though, when both cultures had plateaued with the p24 antigen and the cultured supernatant, we recovered PBS that were complementary to tRNA^Lys,3^. Most probably, the process of reversion for this virus occurred through the formation of the PBS complementary to tRNA^Thr ^followed by the subsequent conversion to a PBS complementary to tRNA^Lys,3 ^which resulted in the high level replication observed for both of these viruses. In contrast, analysis of viruses in which the U5-PBS was complementary to tRNA^Gln,3 ^gave a different pattern. In this case, all of the PBS recovered were complementary to tRNA^Thr^, suggesting that the virus had selected tRNA^Thr ^from the intracellular milieu rather than the starting tRNA(tRNA^Gln,3^) and was now stably using tRNA^Thr ^as the primer for reverse transcription.

## Discussion

The original intent of the experiments was to determine whether HIV-1 would accept tRNA^Gln ^as a primer for initiation of reverse transcription. Our experiments were based on a previous study in which we found that MuLV with a PBS mutated to be complementary to tRNA^Gln,1 ^grew well in tissue culture, even though MuLV prefers to use tRNA^Pro ^as the primer for initiation of reverse transcription [26]. In addition to the viruses with the PBS complementary to tRNA^Gln,1^, we also constructed viruses in which the PBS was complementary to the minor species, tRNA^Gln,3^. Since previous studies have shown that this tRNA is induced in MuLV and HIV-1 infected cells at levels approximately 20 fold over the basal level found in cells [23]. Thus, we expected that HIV-1 might tolerate the selection of tRNA^Gln ^as the primer for reverse transcription. However, it was clear from our studies that viruses with a PBS alone complementary to tRNA^Gln,1 ^or tRNA^Gln,3 ^were unstable and reverted back to use tRNA^Lys,3^. Thus, even though expression of tRNA^Gln,3 ^might be enhanced in HIV-1 infected cells, this tRNA is not a preferred tRNA for selection.

Previous studies from our laboratory and others have found that regions within U5 can be altered in such a way as to facilitate the selection of alternative primers by HIV-1 for reverse transcription [15-20]. A mutation of the region upstream of the PBS (designated the A-loop) so as to be complementary to the anticodon region of certain tRNAs allows these tRNAs to be selected by HIV-1 as the primer for reverse transcription. However, the inclusion of regions within the A-loop that were complementary to tRNA^Gln ^in combination with a PBS complementary to tRNA^Gln ^had substantial effects on the stability and replication of these viruses. Viruses with a PBS and A-loop complementary to tRNA^Gln,1 ^were essentially non-infectious. While viruses in which only the PBS was altered to be complementary to tRNA^Gln,1 ^(the major tRNA^Gln^) were infectious, they reverted back to utilize tRNA^Lys,3 ^following short-term *in vitro *culture. Interestingly, viruses in which the PBS and A-loop were complementary to the minor species of tRNA^Gln,3 ^were infectious albeit at a greatly reduced level compared to the wild type virus. Thus, forcing HIV-1 to use tRNA^Gln,1 ^or tRNA^Gln,3 ^severely reduced the capacity for replication, indicating that this particular tRNA was not available to the virus for primer selection, even for low level of virus replication.

**Figure 3:**
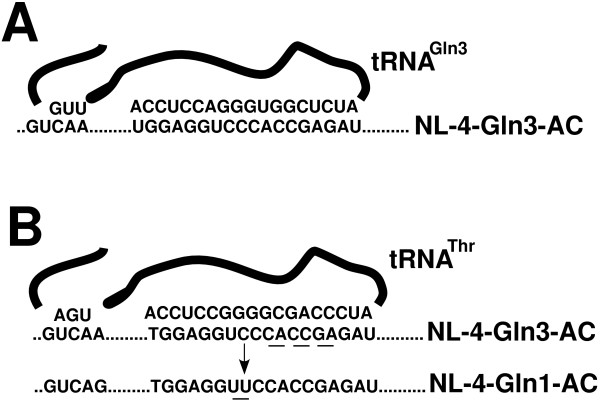
**Sequence complementarity of tRNA^Gln ^and tRNA^Thr ^with mutant proviral genomes**. **Panel A**. Sequence complementary of tRNA^Gln,3 ^with NL-4-Gln3-AC. Depicted is the predicted complementarity between the 3' terminal nucleotides and the PBS and the anticodon of tRNA^Gln,3 ^with the modified A-loop region of NL-4-Gln3-AC. **Panel B**. Complementarity between 3' terminal nucleotides of tRNA^Thr ^with the PBS of NL4-Gln3-AC. Nucleotide differences within the PBS and tRNA^Thr ^are underlined. The anticodon region of tRNA^Thr ^has complementarity with the modified A-loop region of NL-4-Gln3. Additional complementarity between tRNA^Thr ^and the PBS of NL-4-Gln1-AC is also shown. The single nucleotide difference between the PBS is underlined. The resulting GC pair of tRNA^Thr ^and the PBS of NL-4-Gln3-AC should be compensated for by a GU base pair. Note also the predicted complementarity between the anticodon region of tRNA^Thr ^with the modified A-loop region of NL-4-Gln1-AC.

The surprising result of this study was the reversion of viruses with the PBS complementary to tRNA^Gln ^to utilize tRNA^Thr^. How this selection occurred is not clear at this time. Comparison of the PBS sequences between those complementary to tRNA^Gln ^and tRNA^Thr ^revealed considerable homology between the first nine nucleotides as well as the last three nucleotides (Figure 3). Previous studies from our laboratory have shown that the first nine and last three to five nucleotides can facilitate the reverse transcription of HIV-1 in which the PBS was made complementary to alternative tRNAs [27]. It is clear that following selection of tRNA^Thr^ the virus could, through the process of reverse transcription, convert the PBS to be complementary to this tRNA and allow limited growth. Why the virus with a PBS and A-loop complementary to tRNA^Gln,1 ^did not convert to use tRNA^Thr ^is unknown. It is possible that the selection of tRNA^Thr ^is passive, rather than active. Thus, if the virus happens to capture tRNA^Thr^, it will grow, albeit more slowly than the wild type virus. The fact that the process of conversion goes through an intermediate with a PBS complementary to tRNA^Thr ^suggests this tRNA has a greater availability for capture than tRNA^Gln^. Additional studies will be needed to address this possibility.

## Conclusion

In the current study, we have characterized the replication of HIV-1 in which the PBS has been altered to be complementary to tRNA^Gln^. Viruses were constructed in which the PBS or PBS and A-loop were modified to be complementary to either tRNA^Gln,1 ^or tRNA^Gln,3^. All viruses were found to have poor replicative capacity and the PBS was unstable following *in vitro *culture. However, analysis of the PBS from integrated proviruses revealed that a new tRNA, tRNA^Thr ^was preferred by HIV-1 for replication indicating that HIV-1 prefers tRNA^Thr ^as a primer for replication.

The results of our study re-enforces the idea that HIV-1 has preferences for the selection of certain tRNAs for replication. Obviously, the most preferred primer for selection is tRNA^Lys,3^. However, the results from our current and previous studies indicate HIV-1 can tolerate other tRNAs as primers. For example, in a previous study, we found that viruses in which the PBS was mutated to be complementary to tRNA^Trp ^reverted to select tRNA^Met ^as the primer for reverse transcription [28]. Viruses such as those with a PBS and A-loop complementary to tRNA^His ^and tRNA^Lys1,2 ^and tRNA^Glu ^have been generated in our laboratory, suggesting the these tRNAs also are acceptable for selection as primers [15-19,29]. Since HIV-1 can select other tRNAs as the primer for reverse transcription, why HIV-1 does not use these other tRNAs for replication is unknown. It is possible that HIV-1 could have access to several different tRNAs during primer selection. However, under certain circumstances where tRNA^Lys,3 ^is not favored, such as that with proviral genomes with certain A-loop modifications, the virus can select other tRNAs, such as tRNA^Met ^and tRNA^Thr ^as the primer for reverse transcription if sufficient complementarity with the PBS exists. Further understanding of the process and what influences the preference for certain tRNAs will be important to resolve the mechanism of primer selection.

## Materials and methods

### Tissue culture

293T cells were grown in Dulbecco's modified Eagle's medium supplemented with 10% fetal bovine serum (FBS), and SupT1 cells were grown in RPMI 1640 medium supplemented with 15% FBS.

### Construction of mutant proviral genomes

Mutagenesis was performed by using the QuikChange II Site-Directed Mutagenesis Kit (Stratagene) according to the manufacturer's instructions. The PBS sequence in the shuttle vector pUC119PBS [29] was changed to be complementary to the 18 3'-terminal nucleotides of tRNA^Gln3 ^using the primers 5'-TGGAAAATCTCTAGCAGTGGAGGTCCCACCGAGATCTGAAAGCGAAAGGGAAACC-3' and 5'-GGTTTCCCTTTCGCTTTCAGATCTCGGTGGGACCTCCACTGCTAGAGATTTT CCA-3', creating the plasmid pUC-Gln3. pUC-Gln3 was then used as a template to mutate the PBS to be complementary to tRNA^Gln1^, with the primers 5'-CTCTAGCAGTGGAGGTTCCAC CGAGATCTGAAAG-3' and 5'-CTTTCAGATCTCGGTGGAACCTCCACTGCTAGAG-3', resulting in plasmid pUC-Gln1. To create the plasmid pUC-Gln3AC, which contains further mutations in the U5 region complementing the anti-codon loop of tRNA^Gln3^, PUC-Gln3 was used as a template, along with the primers 5'-ACCTCCACTGCTAGAGATTGACTCCACTGACTA AAAGGGTCTGAGG-3' and 5'-CCTCAGACCCTTTTAGTCAGTGGAGTCAATCTCTAGC AGTGGAGGT-3'. Likewise, pUC-Gln1AC with U5 sequence complementary to the anti-codon loop of tRNA^Gln1 ^was made by using PUC-Gln1 as a template, with the primers 5'-CCTCAGACCCTTTTAGTCAGTGGAGTCAGTCTCTAGCAGTGGAGGT-3' and 5'-ACCTCCACTGCTAGAGACTGACTCCACTGACTAAAAGGGTCTGAGG-3'. Subsequently, the *Hpa*I-B*ssH*II fragments of pUC-Gln3, pUC-Gln3AC, pUC-Gln1 and pUC-Gln1AC containing the U5-PBS region were sub-cloned between the *Sma*I and *BssH*II sites of pNL4-3 to form the complete pro-viral clones of pNL4-3-Gln3, pNL4-3-Gln3AC, pNL4-3-Gln1 and pNL4-3-Gln1AC. Sequences of pro-viral clones were verified by DNA sequencing.

### Transfection and analysis of viral infectivity

Plasmids were transfected into 293T cells using the Fugene 6 Transfection Reagent (Roche Molecular Biochemicals, Indianapolis, IN) according to the protocol. Briefly, 2 μg of pro-viral plasmid DNA and 3 μl of Fugene 6 reagent were combined in 100 ul serum free DMEM, and incubated at room temperature for 30 min. The mixture was then added to one well of 6-well plate containing 60% confluent 293T cells in 2 ml fresh medium. The transfections was incubated at 37°C overnight, before replaced with fresh medium, and supernatants were collected after 48 hours and stocked at -80°C in aliquots. Levels of infectious virus (IU/μL) in 293T supernatants were determined using the JC53βL assay as previously described [25,30].

### Infection and maintaining of viral cultures

Virus supernatant containing 250 infectious units were added to 10^6 ^SupT1 cells in 125 μl RPMI supplemented with 2% FBS in a 15 ml Falcon conical tube (BD Bioscience) with caps loosened, and incubated at 37°C for 2 hrs to allow absorption, then transferred to a tissue culture flask containing 10 ml RPMI supplemented with 15% FBS to further culture the infected cells. Every 3–4 days, 8 ml of culture were replaced with 8 ml fresh medium, and supernatants and cell pellets were collected every 7 days and stocked at -80°C. Once the infected SupT1 cultures were found to be cleared of cells, 10^6 ^new SupT1 cells were added to continue the culture.

### DNA sequence analysis of pro-viral U5 and PBS region

High-molecular-weight DNA was isolated from SupT1 cell pellets using the Wizard genomic DNA purification kit (Promega, Madison, WI) according to the manufacturer's instructions. A fragment containing the U5 and PBS regions of the integrated provirus was PCR amplified from the high-molecular weight DNA using primers 5'-CGGAATTCTCTCCTTCTAGCCTCCGCTAGTC-3' and 5'-CCTTGAGCAT GCGATCTACCACACACAAGGC-3'. The PCR products were run on a 1% agarose gel and DNA running approximately 750 bp size were extracted using the Qiagen Gel Purification Kit (Qiagen, Valencia, CA) and sub-cloned into pGEM-T-Easy vector (Promega Madison, WI) according to the protocol. White colonies were picked and grown to produce DNA, which were screened for inserts by EcoRI enzyme digestion. The U5-PBS sequence of TA clones containing the approximately 750 bp inserts were analyzed by automated DNA sequencing, using the primer corresponding to the T7 promoter sequence flanking the multiple cloning site of the vector.

## Competing interests

The author(s) declare that they have no competing interests.

## Authors' contributions

ML, PGE, NN and CDM conceived the studies and ML, PGE and NN performed the experiments. CDM and ML wrote the manuscript.
